# Understanding the P-Loop Conformation in the Determination of Inhibitor Selectivity Toward the Hepatocellular Carcinoma-Associated Dark Kinase STK17B

**DOI:** 10.3389/fmolb.2022.901603

**Published:** 2022-05-10

**Authors:** Chang Liu, Zhizhen Li, Zonghan Liu, Shiye Yang, Qing Wang, Zongtao Chai

**Affiliations:** ^1^ Department of Hepatic Surgery VI, Eastern Hepatobiliary Surgery Hospital, The Second Military Medical University (Navy Medical University), Shanghai, China; ^2^ Department of Biliary Surgery I, Eastern Hepatobiliary Surgery Hospital, The Second Military Medical University (Navy Medical University), Shanghai, China; ^3^ Oncology Department, Xin Hua Hospital Affiliated to Shanghai Jiao Tong University School of Medicine, Shanghai, China; ^4^ Department of Hepatic Surgery, Shanghai Geriatric Center, Shanghai, China

**Keywords:** protein kinase, STK17B, p-loop, molecular dynamics simulation, conformational dynamics

## Abstract

As a member of the death-associated protein kinase family of serine/threonine kinases, the STK17B has been associated with diverse diseases such as hepatocellular carcinoma. However, the conformational dynamics of the phosphate-binding loop (P-loop) in the determination of inhibitor selectivity profile to the STK17B are less understood. Here, a multi-microsecond length molecular dynamics (MD) simulation of STK17B in the three different states (ligand-free, ADP-bound, and ligand-bound states) was carried out to uncover the conformational plasticity of the P-loop. Together with the analyses of principal component analysis, cross-correlation and generalized correlation motions, secondary structural analysis, and community network analysis, the conformational dynamics of the P-loop in the different states were revealed, in which the P-loop flipped into the ADP-binding site upon the inhibitor binding and interacted with the inhibitor and the C-lobe, strengthened the communication between the N- and C-lobes. These resulting interactions contributed to inhibitor selectivity profile to the STK17B. Our results may advance our understanding of kinase inhibitor selectivity and offer possible implications for the design of highly selective inhibitors for other protein kinases.

## Introduction

Protein kinases transfer the γ-phosphate group of ATP to serine, threonine, or tyrosine residues of their substate proteins. This physiological process is also called as phosphorylation. Protein phosphorylation provokes cellular signal transduction cascades associated with cell differentiation, growth, homeostasis, and death (Pearce et al., 2010). Aberrant protein kinase function by either activating mutations or translocations is related with numerous disease states, including cancer, Alzheimer disease, Parkinson’s disease, inflammation, and metabolic disease (Attwood et al., 2021; Cohen et al., 2021). Protein kinase are thus important therapeutic targets for drug discovery. Until now, 71 small-molecule kinase inhibitors have been approved by the FDA in the treatment of cancer and other diseases (Roskoski, 2021).

Despite the inspiring clinical benefits, kinase inhibitors are still encountered an unsurmountable challenge hallmarked by kinase selectivity profile. This is because that the vast majority of protein kinase inhibitors bind to the conserved ATP-binding site, leading to the poor selectivity of kinase inhibitors towards a unique kinase (Wu et al., 2015; Chen et al., 2020; Li C. et al., 2020). For example, Davis et al. (2011) have previously explored the interaction of 72 kinase inhibitors with 442 kinases representing >80% of the human catalytic protein kimome and found that the kinase inhibitor selectivity profile is relatively narrow, with 10%–40% of inhibitors interacting with >60% of kinases, and each inhibitor interacting with more than one kinase. Therefore, developing a promising strategy to discover highly selective inhibitors is an area of intensive research in kinase kinome (Lu et al., 2018, Lu et al., 2019a; Lu and Zhang, 2019).

To achieve inhibitor selectivity, several successful strategies have been reported. Covalent kinase inhibitors are a class of compounds that harbour a reactive, electrophilic warhead, reacting with a nucleophilic cysteine residue at the target site and then forming a stable covalent adduct (Nussinov and Tsai, 2015; Lu and Zhang, 2017; Ni et al., 2020). These covalent inhibitors have pharmacological advantages of high potency and selectivity. For instance, in the double mutant T790M/L858R epidermal growth factor receptor (EGFR), the FDA-approved Osimertinib engages with Cys797 at the ATP-binding site through a covalent bond (Jia et al., 2016; Nussinov et al., 2022). However, in the ATP-binding site, the availability of cysteine residues at the proper position is scarce for most of kinases, rendering the design of covalent inhibitors remaining a challenging task.

**FIGURE 1:**
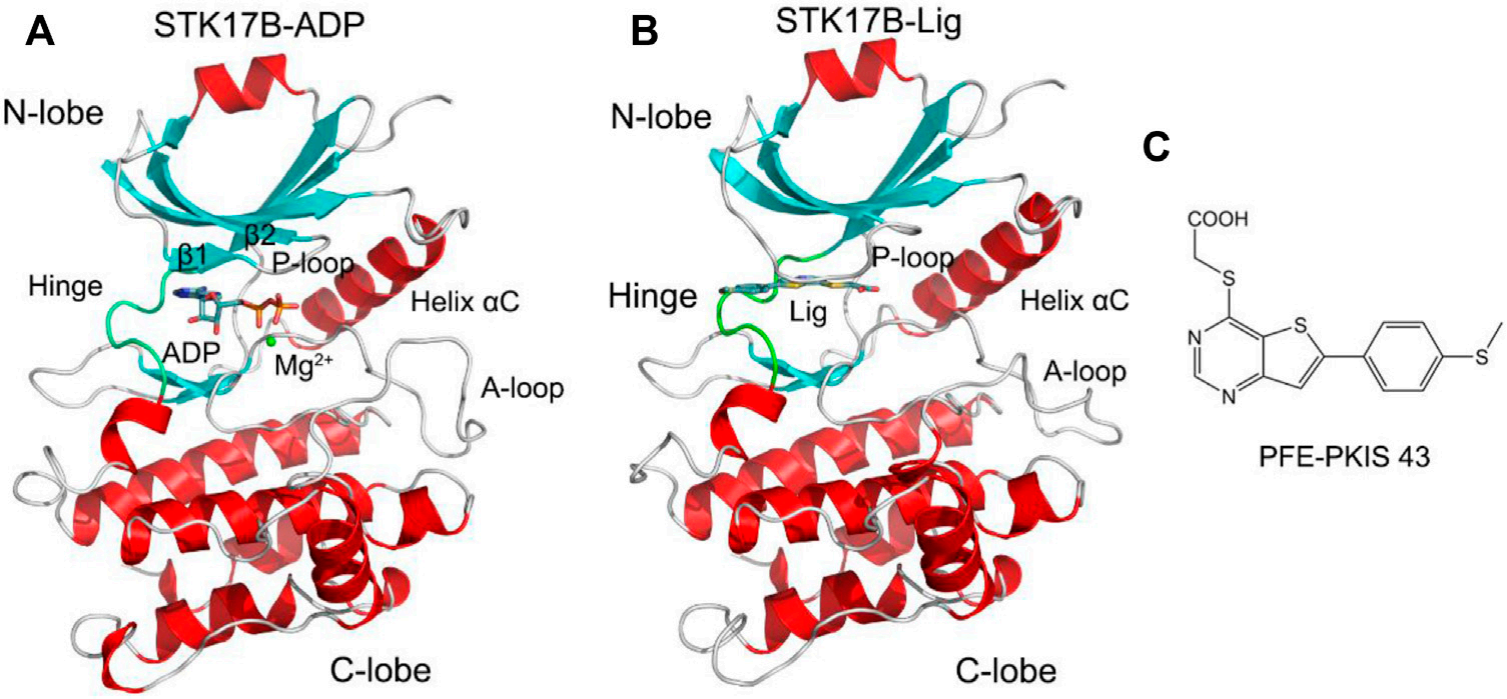
Cartoon representation of STK17B in complex with ADP (PDB ID: 6QF4) **(A)** and the inhibitor PFE-PKIS 43 (PDB ID: 6Y6F) **(B)**. The secondary structural elements of α-helices and β-strands are colored by red and cyan, respectively. The loop including the phosphate-binding loop (P-loop) and the activation loop (A-loop) is colored by gray. The hinge domain is colored by green. ADP and inhibitor are depicted by stick representation. Mg^2+^ ion is shown by a green sphere. **(C)** Chemical structure of the inhibitor PFE-PKIS 43.

Harnessing the sequence differences of ATP-binding site that control inhibitor selectivity has emerged as an alternative. One quintessential example is STK17B, a member of the death-associated protein kinase family of serine/threonine kinases (Pearce et al., 2010). Overexpression of STK17B plays a crucial role in hepatocellular carcinoma and thus, inhibition of STK17B catalytic activity in cells implies clinical utility in the treatment of this malignancy (Lan et al., 2018). The crystal structure of ADP-bound STK17B contains a small N-lobe and a large C-lobe (Figure 1A). The N-lobe is mainly consisted of five β-strands and one catalytic helix αC. The phosphate-binding loop (P-loop) connecting the β1 to the β2 adopts a “U” shape. The C-lobe is largely constituted by helices. The activation loop (A-loop) that control catalytic activity runs along the substrate binding groove. The flexible hinge domain connects the N-lobe to the C-lobe. ADP binds to the cleft between the two lobes located under the P-loop. There are several reported STK17B inhibitors, including quercetin **1**, dovitinib **2**, and benzofuranone **3** (Supplementary Figure S1). However, these are non-selective or modest selective inhibitors toward STK17B. Recently, Picado et al. (2020) reported a cell active STK17B inhibitor, thieno[3,2-d] pyrimidine PFE-PKIS 43 (Figure 1B), which had remarkable potency and selectivity toward STK17B against other homologous protein kinases. A crystal structure of PFE-PKIS 43 complexed with STK17B highlights a unique P-loop flip that interacts with the inhibitor. In addition to the crystal structure of STK17B−PEF-PRIS 43 complex, there are five co-crystal structures of STK17B in complex with different inhibitors previously reported, including EBD (PDB ID: 3LMO), quercetin (PDB ID: 3LM5), UNC-AP-194 probe (PDB ID: 6Y6H), AP-229 (PDB ID: 6ZJF), and dovitinib (PDB ID: 7AKG). Structural superimposition of the five co-crystal structures shows that the P-loop conformation in these structures adopts the ordered β-strands (Supplementary Figure S2), which is different from that in the crystal structure of STK17B−PEF-PRIS 43 complex. However, the conformational dynamics of the P-loop in the STK17B−PEF-PRIS 43 complex remain unexplored.

Here, we performed a multi-microsecond length molecular dynamics (MD) simulation of STK17B in the ligand-free, ADP-bound, or ligand-bound states, to characterize the conformational plasticity of the P-loop and its interplay with the ligand over long time-scales. We collected an overall simulated trajectories of 27 μs, which were conducted in multiple replicates in different states. Coupled with the analyses of principal component analysis (PCA), cross-correlation and generalized correlation motions, secondary structural elements, and community networks, the distinct conformational dynamics of the P-loop in the different states were presented. Our results will advance our understanding of kinase inhibitor selectivity and provide hits for the design of selective inhibitors for other protein kinases.

## Results and Discussion

### System Stability

Based on the available X-ray crystal structures of STK17B, we collected conformational ensembles of μs-length MD simulations. We simulated STK17B in various states (i.e., ligand-free, ATP-bound, or ligand-bound) to explore differences and similarities during MD simulations. For each system, MD simulations were performed in explicit water environment, collecting multiple μs-length trajectories (i.e., 3 replicates of 3 μs each) and yielding a total of sampling of 27 μs. Such a multiple and independent μs-length MD trajectory has been proved efficient for investigating the interdependent conformational plasticity of the kinase domains (i.e., P-loop and A-loop) and their interactions with the ADP or the ligand (Lu et al., 2019b; Zhang et al., 2019; Lu et al., 2021a; Lu et al., 2021b; Maloney et al., 2021; Ni et al., 2021; Hu et al., 2022).

We first monitored the root mean square deviation (RMSD) of the kinase Cα atoms averaged over three replicates for each system. As shown in Supplementary Figure S3, the kinase backbone reached a similar stability in the apo (ligand-free), ADP-bound, and ligand-bound states (i.e., the RMSD reaches 1–1.5 Å). This suggested that upon ADP or ligand binding, the overall stability of the kinase has no significant conformational differences during the simulations.

### Coupled Motions of Kinase Intradomains

The dynamic correlation analysis was carried out to probe the interdependent dynamics among different kinase domains. Two distinct methods, including the traditional Pearson cross-correlation (CC*ij*) and the generalized correlation (GC*ij*), were used to calculate the correlation analysis (Shibata et al., 2020; Liang et al., 2021; Zhang et al., 2022a), which was conducted and averaged over all MD trajectories. The CC*ij* analysis describes the collinear correlation between the two residue Cα atoms (*i* and *j*), reflecting whether they move in the correlated motions (CC*ij* > 0) or in the anti-correlated (CC*ij* < 0) motions. The GC*ij* analysis monitors the degree of correlation between the two residue Cα atoms (*i* and *j*), reflecting how much information of one atom’s positions is provided by that of another atom. The GC*ij* analysis cannot identify correlated or anticorrelated motions of the two atoms, ignoring the elucidation of atom’s motions.

**FIGURE 2:**
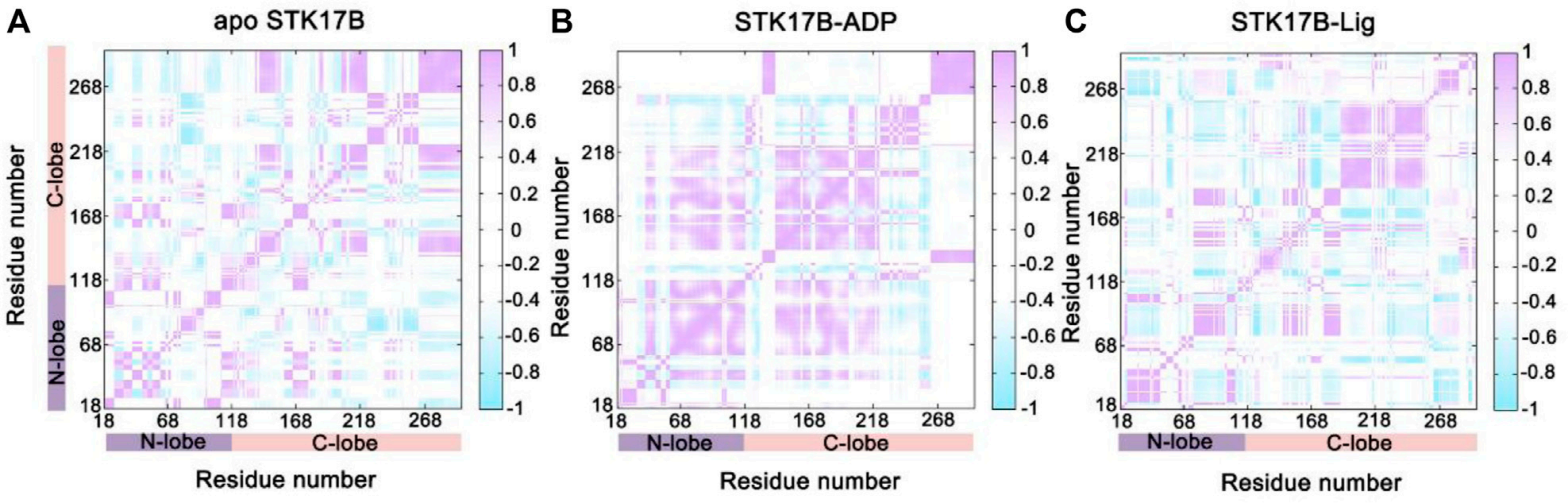
The cross-correlation (CC_*ij*_) matrix of STK17B for the apo **(A)**, ADP-bound **(B)**, and ligand-bound **(C)** systems. The correlated motions are colored by violent (CC_*ij*_ > 0), while the anti-correlated motions are colored by cyan (CC_*ij*_ < 0). Color scales are shown at the right. The CC_*ij*_ values with an absolute correlation coefficient of <0.4 are colored by white for clarity.

**FIGURE 3:**
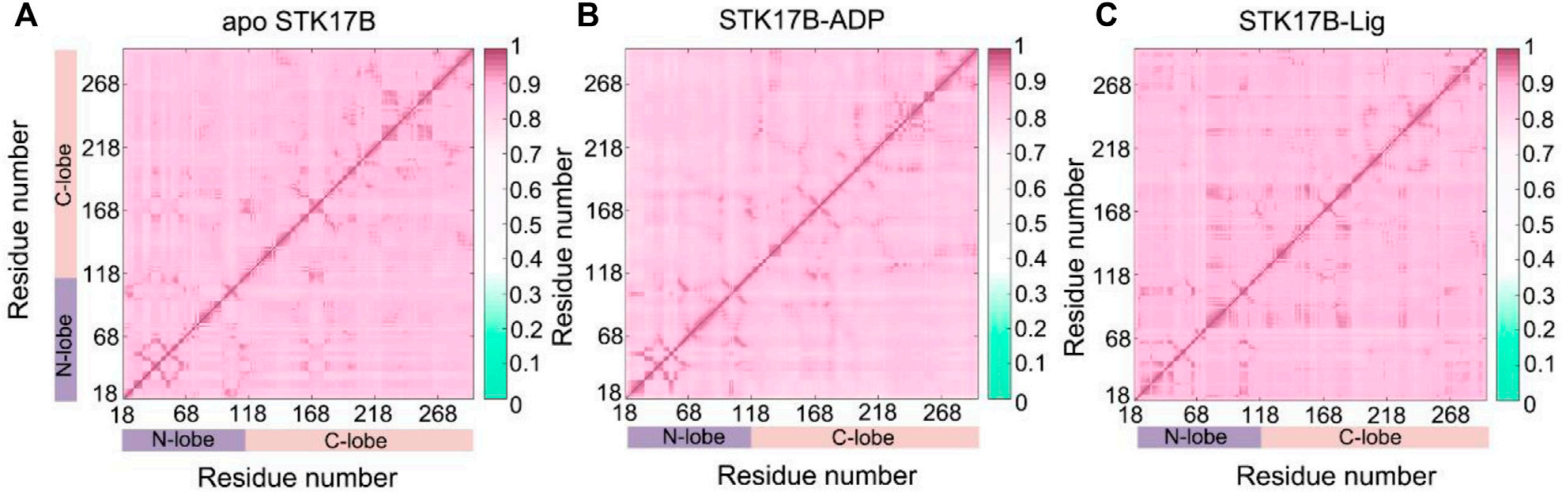
The generalized correlation (GC_*ij*_) matrix of STK17B for the apo **(A)**, ADP-bound **(B)**, and ligand-bound **(C)** systems. Color scales are shown at the right.

The CC*ij* matrix of STK17B that is represented by a two-by-two plot of the Cα CC*ij* coefficients reveals a conserved pattern of correlated/anticorrelated motions in all apo, ADP-bound and ligand-bound states (Figure 2). The N-lobe containing the P-loop (residues 40–47) and C-lobe shows anticorrelated motions, which is also observed on other protein kinases such as anaplastic lymphoma kinase (ALK) (Liang et al., 2021), BCR-ABL (Zhang et al., 2022a) and epidermal growth factor receptor (EGFR) (Qiu et al., 2021). This suggests that the opposite movement of the N- and C-lobes favours the “open or closed” conformational transition of the nucleotide binding site underlying ADP/ATP and substrate binding. In addition, the difference matrix of ADP- and ligand-bound states using the apo state as the reference indicates that the opposite movement of the N- and C-lobes was stronger in the ADP-bound state than that in the ligand-bound state (Supplementary Figure S4). The GC*ij* analysis was further used to unravel the global dependencies of the protein kinase domain motions (Figure 3). Like the CC*ij* matrix, the GC*ij* matrix of the STK17B in the apo, ADP-bound and ligand-bound states showed a high degree of correlations between the N-lobe and the C-lobe. However, the protein in the ligand-bound system had a slightly higher correlations than that in the ADP-bound and apo systems, which was further supported by the difference matrix of ADP- and ligand-bound states using the apo state as the reference (Supplementary Figure S5). This result indicated that ligand binding induced an enhanced motions of protein kinase domains.

### Local Motions and Conformational Dynamics

In order to unravel the predominant collective motions of different STK17B states and capture their essential degrees of freedom, we conducted principal component analysis (PCA) of STK17B in the apo, ADP-bound, and ligand-bound states. Based on the PCA, the first two principal modes of motion (i.e., principal components 1 and 2, PC1 and PC2) provide information regarding to the large-amplitude motions of different STK17B states, which represent their functional dynamics (Masterson et al., 2011; Chen et al., 2019; Chen et al., 2021; He et al., 2021; Okeke et al., 2021; Rehman et al., 2021). In PCA, we selected all simulated trajectories for each system and subjected to RMS-fit to the same initial structure to rule out the translational and rotational motions of the protein.

**FIGURE 4:**
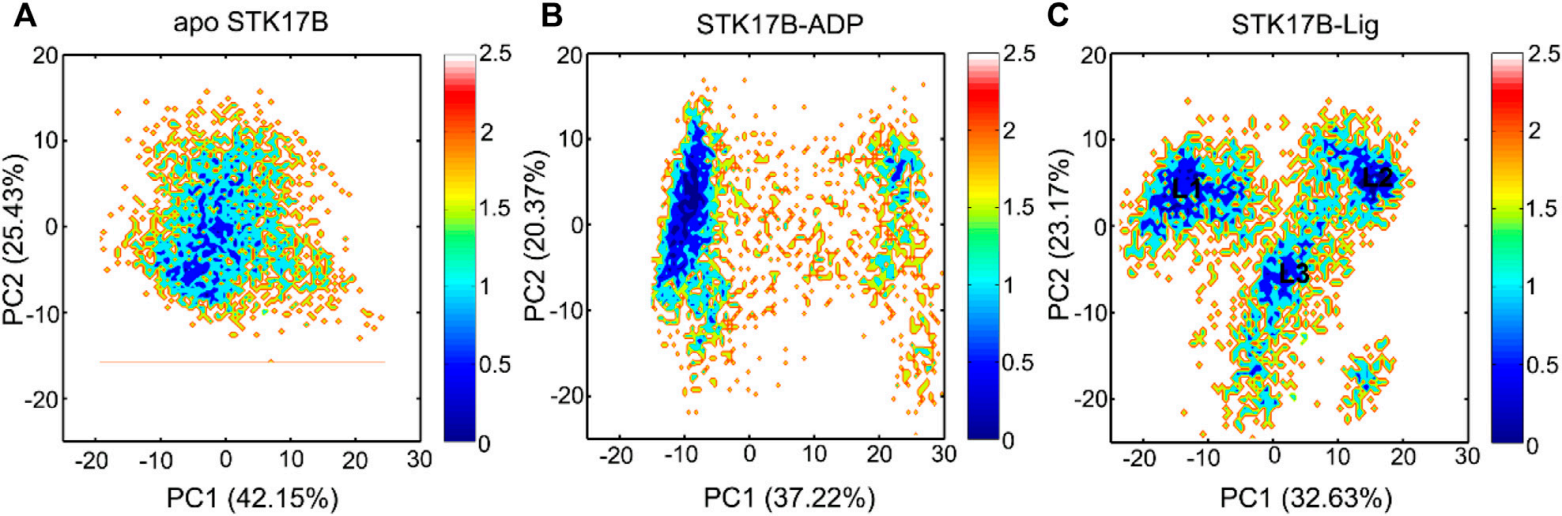
The free energy landscape of the first and second principal components (PC1 and PC2) for the apo **(A)**, ADP-bound **(B)**, and ligand-bound **(C)** systems. The unit of free-energy values is kcal/mol.

As shown in Figure 4A, the apo protein sampled a confined distribution of conformations. Addition of ADP largely changed PC1, but did not change PC2 (Figure 4B), indicating that the protein kinase had increased dynamics in response to ADP binding. More remarkably, in the ligand-bound system (Figure 4C), both PC1 and PC2 were enlarged compared to the apo and ADP-bound systems. This observation suggested that the ligand binding induced more enhanced conformational dynamics of STK17B, which was consistent with the GC*ij* analysis. We further extracted the most represented conformation from each cluster in the ligand-bound state (L1–L3). As shown in Supplementary Figure S6, structural overlapping of the three most represented conformations showed that the P-loop and A-loop in the ligand-bound STK17B underwent obvious conformational changes. Indeed, previous MD simulations of protein kinase A (PKA) also indicated that ligand binding induced global transitions in the catalytic domain of PKA (Hyeon et al., 2009), supporting our MD simulation results of ligand-bound STK17B.

**FIGURE 5:**
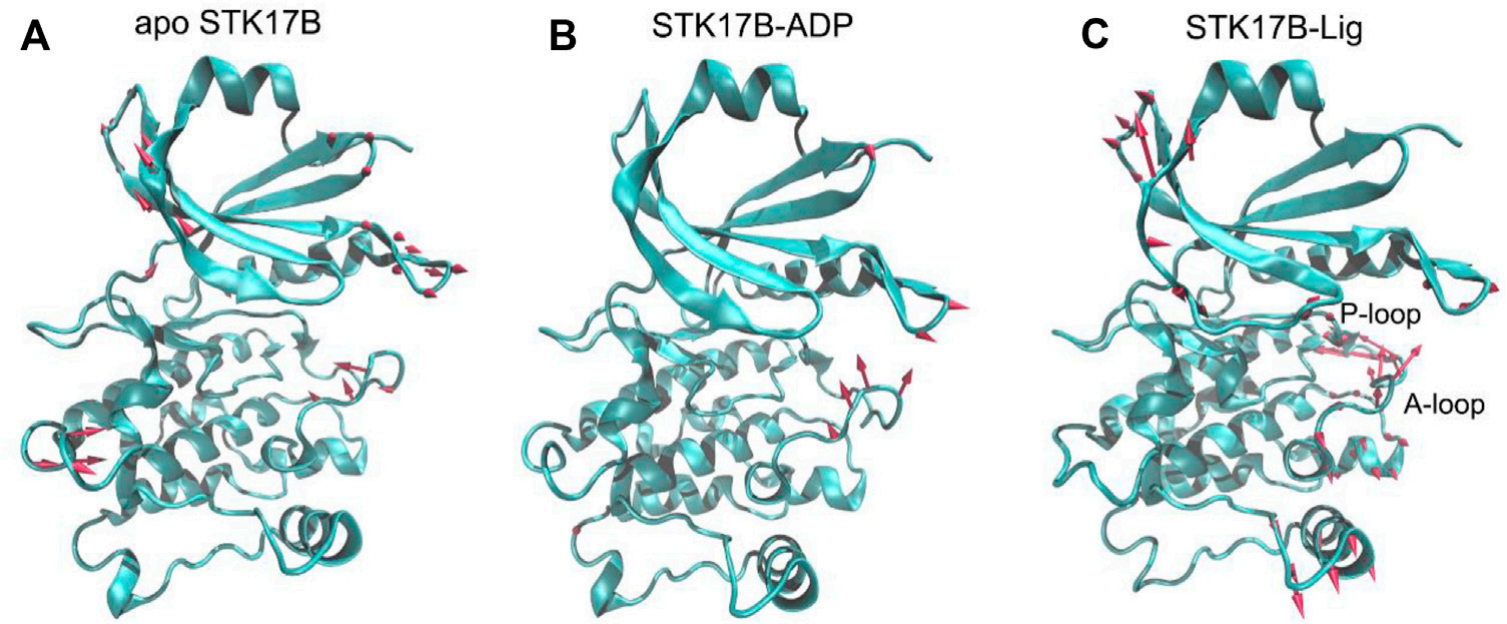
The motion of the first principal component (PC1) for the apo **(A)**, ADP-bound **(B)**, and ligand-bound **(C)** systems. The red arrows represent the direction, with length proportional to the intensity of the motion.

The conformational landscapes of different STK17B states based on the PCA results implied that STK17B was more dynamics in the presence of ligand. To further validate this hypothesis, the PC1 of the STK17B in the three different states was visualized on the 3D structure (Figure 5). The red arrows show the direction of residue motions, with the length proportional to the intensity of the motion. Remarkably, the ligand binding (Figure 5C) triggered more dynamic movement of P-loop and A-loop than the apo (Figure 5A) and the ADP-bound (Figure 5B) systems. For instance, no motion of the P-loop, but a weak motion of the A-loop was observed in both the apo and ADP-bound systems. In agreement with the PCA results, both the P-loop and the A-loop of STK17B in the presence of ligand were highly flexible, which may determine the selectivity profile of ligand to the STK17B.

### Secondary Structural Analysis of the Phosphate-Binding Loop

**FIGURE 6:**
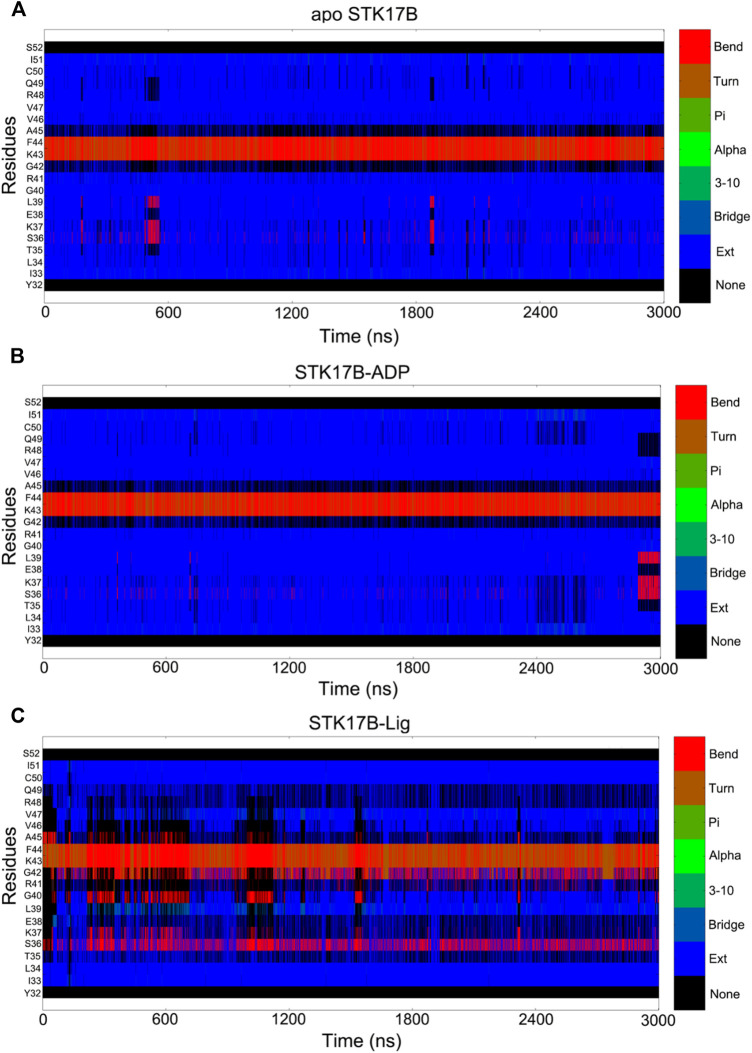
Secondary structural element analysis as a function of simulation time for residues Tyr32 to Ser52 in the apo **(A)**, ADP-bound **(B)**, and ligand-bound **(C)** systems as calculated using the defined secondary structure of proteins (DSSP) method.

To further reveal the different secondary structures of the P-loop in the three different STK17B states, the defined secondary structure of proteins (DSSP) (Lei et al., 2019) method was used to analyse the secondary structural elements of residues Tyr32−Ser55. Figure 6 shows the secondary structural profile of residues Tyr32−Ser55 for the three systems. In both the apo (Figure 6A) and ADP-bound (Figure 6B) systems, the residues Ile33−Arg41 and Val46−Ile51 formed two extended strands (β1 and β2) and residues Gly42−Ala45 at the P-loop adopted the bend conformation. These secondary structural elements of the β1, P-loop and β2 in the apo and ADP-bound states are consistent with the typical protein kinases at the corresponding position. In sharp contrast, in the ligand-bound state (Figure 6C), the secondary structural conformation of the β-strand in the residues Ile33−Arg41 and Val46−Ile51 was disturbed, especially the residues Ile33−Arg41 in the disordered conformation. Together, DSSP results indicated that the conformational changes of residues Ile33−Arg41 induced by the ligand binding may have an important role in the control of inhibitor selectivity to the STK17B.

### Community Network Analysis

We next performed community network analysis to reveal the altered community networks of STK17B in the apo, ADP-bound, and ligand-bound states. The whole simulated trajectories were selected for community network analysis. The two Cα atoms within a cut-off distance of 4.5 Å that has an occupation time >75% of simulation time were classified into the same community (Sethi et al., 2009; Liang et al., 2020; Li et al., 2021a; Foutch et al., 2021; Tian et al., 2021). Each community was represented by coloured circles whose size is related to the number of residues it includes. The strength of the two communities was represented by the width of sticks that connect inter-communities.

**FIGURE 7:**
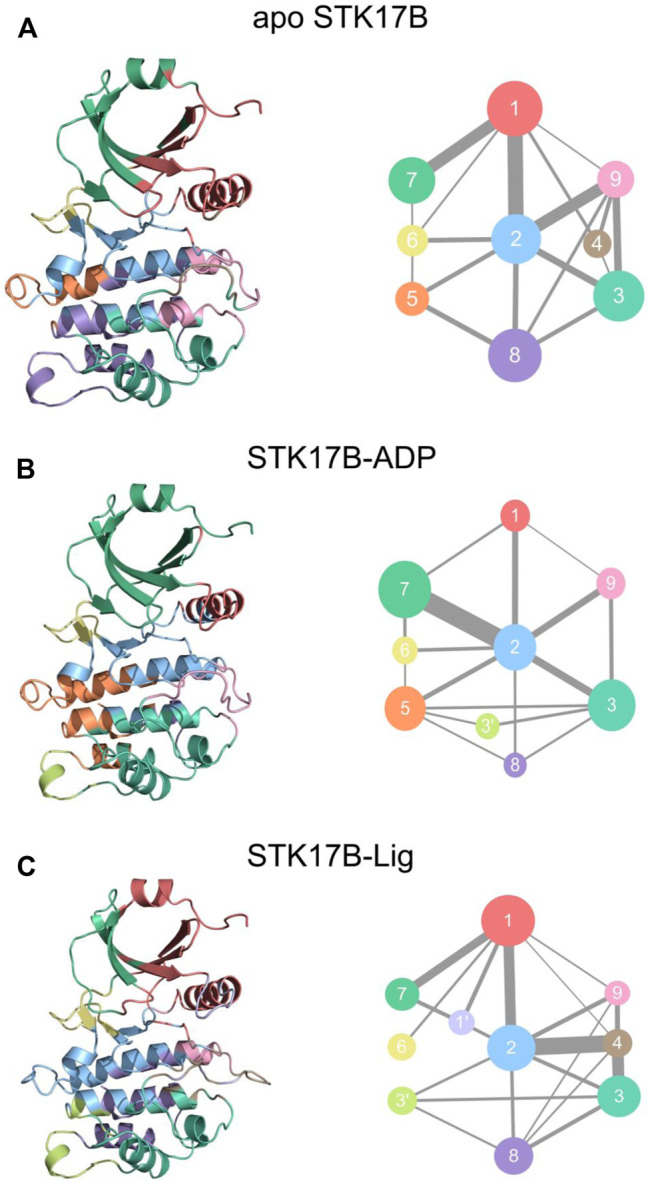
The community networks for the apo **(A)**, ADP-bound **(B)**, and ligand-bound **(C)** systems. The communities are shown as circles with different colors. The edges represent the connections among communities and the width is related to the intensity of the connections among communities.

Figure 7 shows the communities of different STK18B states. In the apo system (Figure 7A), there has nine communities. The community **1** contains the P-loop, the helix αC, and the β3-β5. The community **2** consists of the helix αD and the β6-β7. The community **9** largely includes the A-loop. There was the existence of strong connection between the community **1** and community **2** and between the community **1** and community **9**. In contrast, the communication between the community **1** and community **9** was weak. This observation indicated that there was no information flow between the P-loop and the A-loop in the apo system. In the ADP-bound system (Figure 7B), the community **1** diminished, which only consists of the helix αC. The sizes of the community **2** and community **9** in the ADP-bound system were similar to those in the apo system. However, the information flow that connects between the community **1** and community **2** and between the community **1** and community **9** was markedly weaker in the ADP-bound system than in the apo system. This indicated that upon ADP binding to the STK17B, the inter-domain interaction between the P-loop in the N-lobe and the helix αD in the C-lobe became weaken compared to the apo system. In the ligand-bound system (Figure 7C), the community **1** was enlarged compared to the ADP-bound systems, which was the same with the apo system. The community **1** in the ligand-bound systems consists of the P-loop, the helix αC, and the β3-β5. More significantly, the communication between the community 1 and community **2** in the ligand-bound system was enhanced compared to the ADP-bound system, with the strength resembling to the apo system. This observation suggested that upon ligand binding to the ADP-bound site, the information flow between the P-loop in the N-lobe and the helix αD in the C-lobe became stronger compared to the ADP-bound system. This enhanced interactions between the two lobes may promote inhibitor binding and selectivity to the STK17B.

### Comparative Binding Modes

**FIGURE 8:**
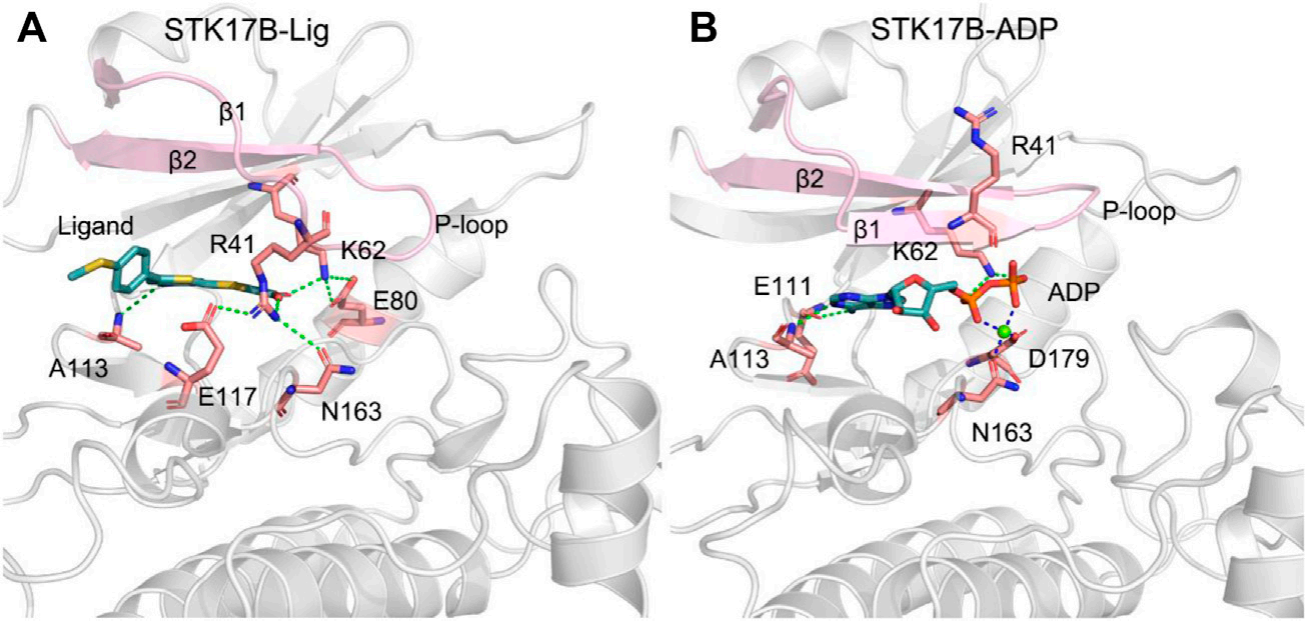
The most representative structural complexes of ligand-bound **(A)** and ADP-bound **(B)** STK17B. The β1 and β2 and the P-loop are colored by pink. Hydrogen bonds or salt bridges are shown by green dotted lines. Coordinated bonds are shown by blue dotted lines.

Community network analysis implied the strong interactions between the N- and C-lobes in response to the ligand binding. To further elucidate the conformational arrangement of the two lobes of the protein kinase and the detailed interactions of ADP and the ligand with the STK17B, the most representative conformation of the STK17B-ligand and STK17B-ADP complexes was obtained using the cluster analysis of the three simulated trajectories (Liu et al., 2018; Xie et al., 2019). As shown in Figure 8A, in the ligand-bound state, there was a significantly disordered conformation of the P-loop, especially the β1, which was in good agreement with the DSSP results. Owing to the disordered P-loop conformation, the Arg41 at the β1 was flipped into the ADP-binding site and formed hydrogen bonding or salt bridge interactions with the residues Glu117 and Asn163 at the C-lobe and the carboxylic acid of the ligand. The hydrogen bonding occupation percentage was summarized in the Supplementary Table S1. These interactions promoted the strong communication between the N- and C-lobes, which contributed to increase the selectivity profile of the ligand to the STK17B. Simultaneously, the carboxylic acid of the ligand also interacted with the catalytic residue Lys62 through a salt bridge. Lys62 in turn formed salt bridge interactions with the Glu80 at the helix αC. In addition, the N1 of the thieno[3,2-d]pyrimidine formed a hydrogen bond with the amide backbone of Ala113 at the hinge domain. In contrast, in the ADP-bound state (Figure 8B), the β1 and β2 formed two anti-paralleled strands, which was consistent with the DSSP results. Owing to the ordered P-loop conformation, the Arg41 at the β1 was protruded into the solvent and had no interactions with the C-lobe, which was markedly different from that in the ligand-bound state. In the hinge domain, the backbone of residues Glu111 and Ala113 formed two hydrogen bonds with the adenine moiety of ADP. The hydrogen bonding occupation percentage was summarized in the Supplementary Table S2. The catalytic residue Lys62 formed salt bridges with the α- and β-phosphate moieties of ADP and the Mg^2+^ ion was coordinated with the α- and β-phosphate moieties, the carboxylic moiety of Asp179, and the carbonyl moiety of Asn163. Collectively, the comparative binding modes of ADP and the ligand with the STK17B highlighted that the unique *p* conformation induced by the ligand binding played a determined role in the increased selectivity of the ligand to the protein kinase. Given that the important role of the salt bridge interactions between the carboxylic acid moiety of the ligand and Arg41, it is advisable to retain the carboxylic acid moiety in the future drug design toward STK17B.

## Conclusion

In the present study, the collective sampling of 27 μs MD simulations, coupled with the PCA, correlated motion analysis, DSSP, and community network analysis, revealed the effect of the conformational dynamics of the P-loop on the inhibitor selectivity profile to the STK17B. Ligand binding contributed to the increase of the conformational plasticity of the STK17B. Compared to the apo and ADP-bound STK17B, the P-loop, especially the β1, adopted the disordered conformation in the presence of the ligand. This unusual P-loop conformation rendered the residue Arg41 at the β1 flipping into the ADP-binding site and interacted with the carboxylic acid moiety of the ligand and residues Glu117 and Asn163 the C-lobe. These interactions in the ligand-bound state enhanced the information flow between the N- and C-lobes as observed by the community network analysis, which played an essential role in the control of the inhibitor selectivity to the STK17B. Owing to the importance of the salt bridge interactions between the carboxylic acid moiety of the ligand and Arg41 in the maintenance of the unique, disordered P-loop conformation, the carboxylic acid moiety is suggested to retain in the future drug design toward STK17B. These results shed light on the structural basis of the selectivity of the inhibitor to the STK17B, which may be useful for the design of highly selective inhibitors to other protein kinases.

## Materials and Methods

### System Preparation

The co-crystal structures of STK17B in complex with ADP (PDB ID: 6QF4) (Lieske et al., 2019) or PFE-PKIS 43 (PDB ID: 6Y6F) (Picado et al., 2020) were respectively downloaded from the Protein Data Bank (PDB). The missing residues E191−E194 in the 6QF4 and C187−I195 in the 6Y6F at the A-loop were modelled using the MODELLER program (Webb and Sali, 2014). The ADP molecule in the 6QF4 was removed to serve as the ligand-free STK17B (apo STK17B).

The force field parameters for ADP and Mg^2+^ were obtained from the AMBER parameter database (www.amber.manchester.ac.uk) and the generalized AMBER force field (GAFF) (Wang et al., 2004) was used for PFE-PKIS 43. Partial changes for PFE-PKIS 43 were computed using the RESP HF/6-31G* method (Bayly et al., 1993) through the antechamber module in AMBER 18 (Case et al., 2005) and Gaussian 09 program. The AMBER ff14SB (Maier et al., 2015) force field was used for the protein and the TIP3P model was used for water molecules (Jorgensen et al., 1983). The three simulated systems were embedded in a truncated octahedron TIP3P explicit water box with a boundary of 10 Å, while counterions Na^+^ were added to neutralize the total charge. Then, 0.15 mol/L NaCl were added to simulate the physiological environment.

### Molecular Dynamics Simulations

MD simulations were carried out using the AMBER 18 program (Case et al., 2005). Two rounds of minimizations of the three simulated systems were performed, including the steepest descent and conjugate gradient algorithms. This simulation protocol has also been employed in recent studies of protein conformational dynamics (Lu et al., 2019c; An et al., 2021; Liu et al., 2021; Zhang et al., 2022b). Then, each system was heated up from 0 to 300 K within 1 ns of MD simulations in the canonical ensemble (NVT), imposing position restraints of 100 kcal/mol·A^2^ on the solute atoms. Finally, three replicas of independent 3 μs simulations were performed with random velocities under isothermal isobaric (NPT) conditions. An integration time step of 2 fs was used. The SHAKE algorithm was used to constrain all bond lengths involving hydrogen atoms (Ryckaert et al., 1977). The particle mesh Ewald (PME) method was used to treat with the long-range electrostatic interactions (Darden et al., 1993), while a 10 Å non-bonded cut-off was used for the short-range electrostatics and van der Waals interactions.

### Principal Component Analysis

Principal component analysis (PCA) has been widely used to elucidate large-scale collective motions of biological macromolecules during MD simulations (Li et al., 2020b; Li et al., 2021b; Feng et al., 2021), which can transform a series of potentially coordinated observations into orthogonal vectors to capture large-amplitude motions. Among these vectors, the first two principal component (named PC1 and PC2) provide the dominant motions during MD simulations. In PCA, PCs were generated based on coordinate covariance matrix of Cα atoms in the STK17B protein and these collected frames were all projected on the PC1 and PC2.

### Generalized Correlation Analysis

Generalized correlation (GC_*ij*_) analysis was performed to monitor the correlated motions of residues (He et al., 2022; Wang et al., 2022; Zhuang et al., 2022). To describe that how much information of one atom was provided by another atom, Mutual Information (MI) was calculated using the Eq. 1:

$$MI\left[x_{i},x_{j}\right]=\iint p\left(x_{i},x_{j}\right)\;ln\frac{p\left(x_{i},x_{j}\right)}{p\left(x_{i}\right)p\left(x_{j}\right)}dx_{i}dx_{j}$$ (1)

The equation can be calculated using the known measure of entropy as the Eq. 2:

H[x]=∫p(x) ln p(x)dx (2)

The correlation between pairs of atoms $x_{i}$ and $x_{j}$ can be calculated using the marginal Shannon entropy $H\left[x_{i}\right]$ , $H\left[x_{j}\right]$ , and the joint entropy term $H\left[x_{i},x_{j}\right]$ as the Eq. 3:

$$MI\left[x_{i},x_{j}\right]=H\left[x_{i}\right]+H\left[x_{j}\right]-H\left[x_{i},x_{j}\right]$$ (3)

The $MI\left[x_{i},x_{j}\right]$ values can be further normalised to obtain the normalised generalised correlation coefficients ( $GC_{ij}$ ) as the Eq. 4:

$$GC_{ij}\;={\left\{1-e^{-\frac{2MI\left[x_{i},x_{j}\right]}{d}}\right\}}^{-\frac{1}{2}}$$ (4)

where d represents the dimensionality of $x_{i}$ and $x_{j}$ .

### Cross-Correlation Analysis

Based on Pearson coefficients between the fluctuations of the Cα atoms, the cross-correlation matrix (*CC*_*i*j_) was calculated to describe the coupling of the motions between the protein residues (Li et al., 2020b; Aledavood et al., 2021; Hernández-Alvarez et al., 2021; Wang et al., 2021). *CC*_*ij*_ was computed using the following Eq. 5,

$$C\left(i,j\right)=\frac{c\left(i,j\right)}{c{\left(i,i\right)}^{1/2}c{\left(j,j\right)}^{1/2}}$$ (5)

The positive *CC*_*ij*_ values indicate the two atoms *i* and *j* moving in the same direction, whereas the negative *CC*_*ij*_ values indicate the anti-correlated motions between the two atoms *i* and *j*.

### Community Network Analysis

Community network was analyzed to uncover the inter-community interactions using the Network View plugin in VMD (Sethi et al., 2009; Marasco et al., 2021). In this analysis, the Cα atoms in the STK17B were selected as nodes to represent their corresponding residues. Edges were described between nodes whose distances are within a cut-off of 4.5 Å occupying >75% of simulation time. The edge between nodes was calculated using the Eq. 6:

$$d_{i,j}=-\log\left(\left|C_{i,j}\right|\right)$$ (6)

where *i* and *j* represent the two nodes.

## Data Availability

The original contributions presented in the study are included in the article/Supplementary Material, further inquiries can be directed to the corresponding authors.
