# Supplementary material for: Understanding the P-Loop Conformation in the Determination of Inhibitor Selectivity Toward the Hepatocellular Carcinoma-Associated Dark Kinase STK17B
Source: Front Mol Biosci. 2022 May 10;9:901603. doi: 10.3389/fmolb.2022.901603 (PMC9127184; doi:10.3389/fmolb.2022.901603)
Supplement: Supplementary file 1 [file DataSheet1.docx]

**Figure S1:** Chemical structures of STK17B inhibitors.


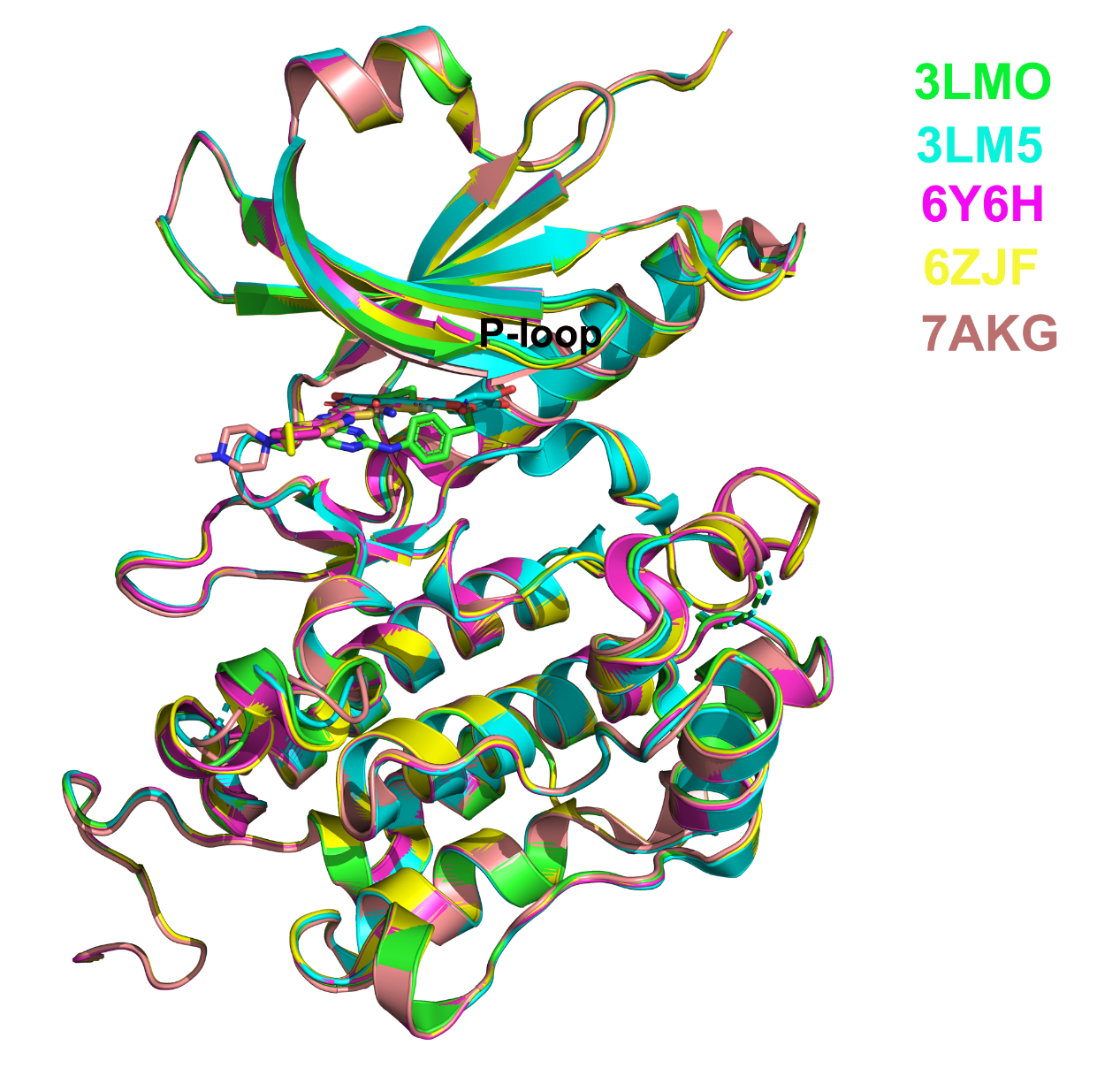


**Figure S2:** Structural superimposition of the five co-crystal structures of STK17B in complex with EBD (PDB ID: 3LMO), quercetin (PDB ID: 3LM5), UNC-AP-194 probe (PDB ID: 6Y6H), AP-229 (PDB ID: 6ZJF), and dovitinib (PDB ID: 7AKG) shows that the P-loop conformation in these structures adopts the ordered β-strands


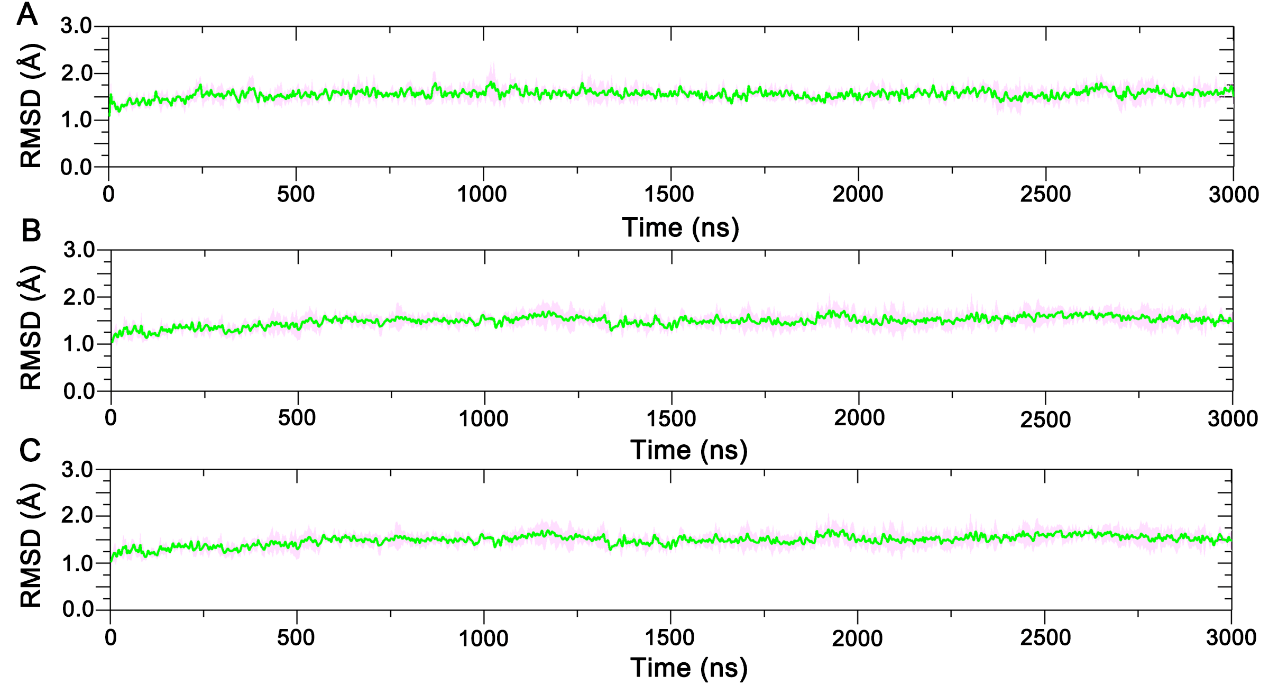


**Figure S3:** The root-mean-square deviation (RMSD) profiles based on the C*α* atoms of STK17B based using all frames of the 3 independent MD simulations for the apo (A), ADP-bound (B), and ligand-bound (C) systems. The SEM error bars show RMSD for 3 replicas related to the average value.


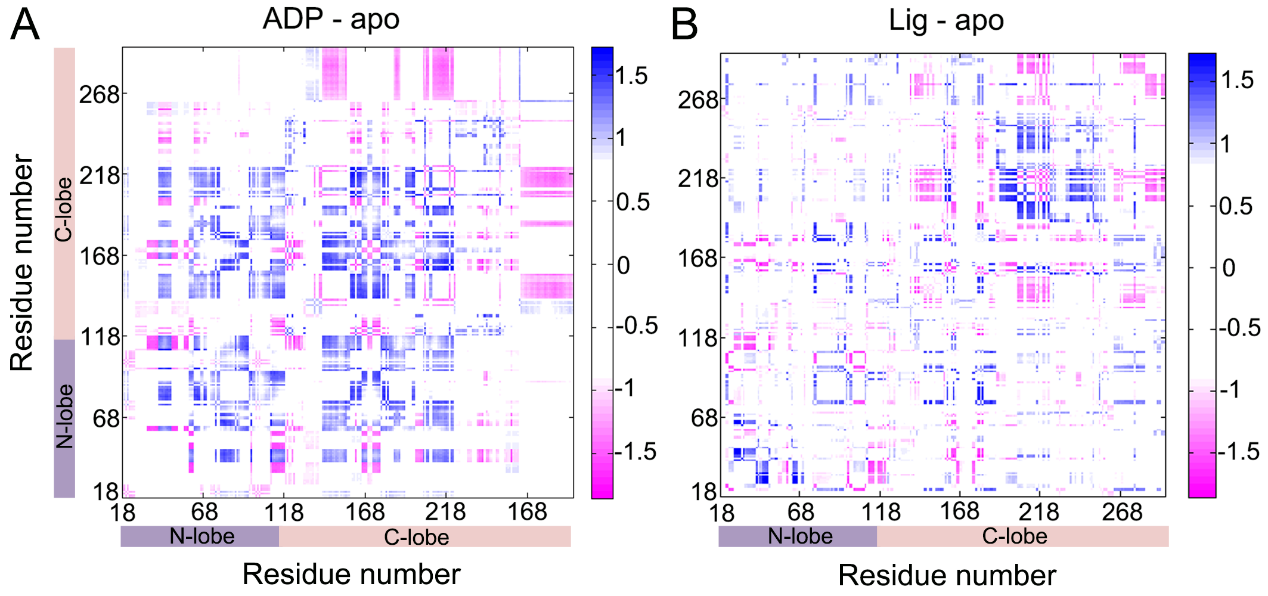


**Figure S4:** (A) The difference of the cross-correlation matrix of STK17B between the ADP-bound state and the apo state. (B) The difference of the cross-correlation matrix of STK17B between the ligand-bound state and the apo state. The apo state is used as the reference.


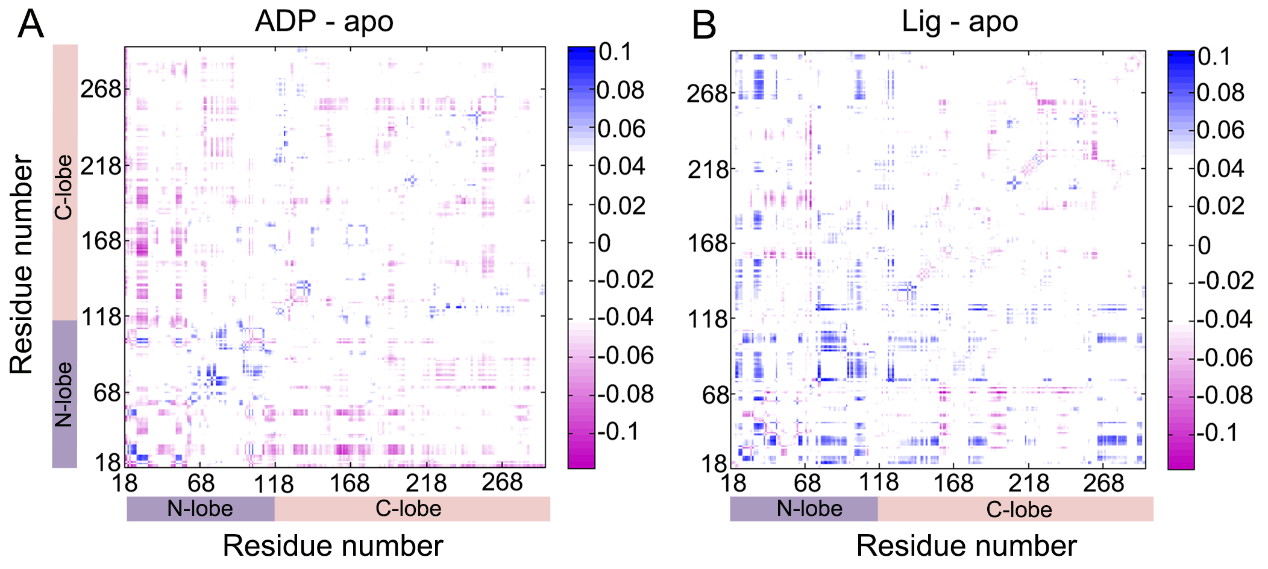


**Figure S5:** (A) The difference of the generalized correlation matrix of STK17B between the ADP-bound state and the apo state. (B) The difference of the cross-correlation matrix of STK17B between the ligand-bound state and the apo state. The apo state is used as the reference.


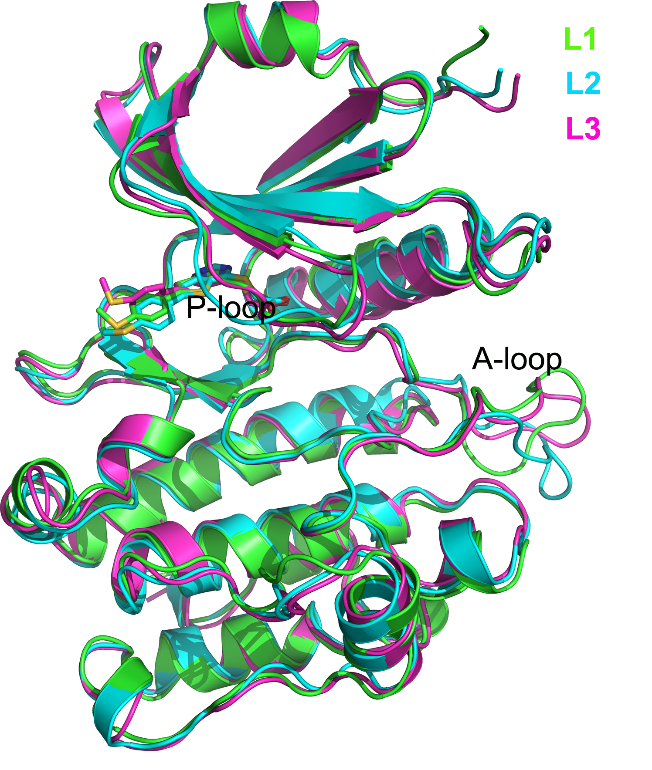


**Figure S6:** Structural overlapping of the three most represented conformations (L1-L3) from the ligand-bound state based on the PCA results.

**Table S1:** Key hydrogen bonding interactions in the ligand-bound state

| Acceptor | Donor | Frac |
| --- | --- | --- |
| Ligand | Ala113 | 93.41% |
| Ligand | Arg41 | 78.73% |
| Ligand | Lys62 | 80.47% |
| Glu80 | Lys62 | 98.93% |
| Glu117 | Arg41 | 73.23% |
| Asn163 | Arg41 | 61.50% |

**Table S2:** Key hydrogen bonding interactions in the ADP-bound state

| Acceptor | Donor | Frac |
| --- | --- | --- |
| Glu111 | ADP | 93.57% |
| ADP | Ala113 | 72.75% |
| ADP-O2A | Lys62 | 83.88% |
| ADP-O2B | Lys62 | 70.73% |
